# The different roles of selective autophagic protein degradation in mammalian cells

**DOI:** 10.18632/oncotarget.5776

**Published:** 2015-09-22

**Authors:** Da-wei Wang, Zhen-ju Peng, Guang-fang Ren, Guang-xin Wang

**Affiliations:** ^1^ Department of Biochemistry and Molecular Biology, School of Medicine, Shandong University, Jinan, Shandong, China; ^2^ Medical Institute of Paediatrics, Qilu Children's Hospital of Shandong University, Jinan, Shandong, China

**Keywords:** autophagy, protein, degradation, modification, autophagy receptor

## Abstract

Autophagy is an intracellular pathway for bulk protein degradation and the removal of damaged organelles by lysosomes. Autophagy was previously thought to be unselective; however, studies have increasingly confirmed that autophagy-mediated protein degradation is highly regulated. Abnormal autophagic protein degradation has been associated with multiple human diseases such as cancer, neurological disability and cardiovascular disease; therefore, further elucidation of protein degradation by autophagy may be beneficial for protein-based clinical therapies. Macroautophagy and chaperone-mediated autophagy (CMA) can both participate in selective protein degradation in mammalian cells, but the process is quite different in each case. Here, we summarize the various types of macroautophagy and CMA involved in determining protein degradation. For this summary, we divide the autophagic protein degradation pathways into four categories: the post-translational modification dependent and independent CMA pathways and the ubiquitin dependent and independent macroautophagy pathways, and describe how some non-canonical pathways and modifications such as phosphorylation, acetylation and arginylation can influence protein degradation by the autophagy lysosome system (ALS). Finally, we comment on why autophagy can serve as either diagnostics or therapeutic targets in different human diseases.

## INTRODUCTION

Autophagy is an evolutionarily conserved eukaryotic process that can be initiated in response to both external and intracellular factors, including amino acid starvation [1], growth factor withdrawal [2], endoplasmic reticulum (ER) stress [3], hypoxia [4], oxidative stress [5], pathogen infection [6], and organelle signaling [7, 8], which are beneficial to cell survival under adverse conditions. Autophagy is precisely regulated by many different proteins. The autophagy-related gene (ATG) family provides the infrastructure for autophagy; until recently, 40 ATG genes had been identified, primarily through genetic studies in yeast [9]. ATG proteins are classified according to their functions into four groups: the uncoordinated-51-like protein kinase (ULK) complex, the ATG9-ATG18 complex, the class III phosphatidylinositol 3-kinase (PI3K) complex, and two ubiquitin-like protein (UBL) conjugation systems [10]. The ULK complex controls the early steps of autophagosome formation under certain induction signals such as nutrient deprivation, hypoxia and ER stress by recruiting the ATG9-ATG18-ATG2 complex to form phagophore assembly sites (PAS) [11, 12]. ATG9, the only known ATG membrane protein, together with ATG2 and ATG18 form a recycling system that provides the lipids for autophagosome production and growth [12]. The class III PI3K complex, whose members include ATG14, Beclin1, Vps34 and Vps15, functions in the nucleation phase of autophagy [12]. ATG14 facilitates the complex formation by recruiting Beclin1, Vps34 and Vps15 and targets the formed class III PtdIns3K complex in the PAS [13]. The UBL conjugation cascade is composed of the E1 enzyme ATG7, two E2 enzymes (ATG10 and ATG3) and two UBLs (ATG8 and ATG12) [10]. ATG12 is conjugated to a lysine residue in ATG5 via the ATG7-ATG10 cascade, ultimately forming an oligomeric ATG12-ATG5-ATG16 complex that promotes the conjugation of the carboxy-terminal Gly residue of ATG8 to phosphatidylethanolamine (PE) via ATG3 and ATG7 [10, 12].

Unfolded or misfolded cellular proteins are generally degraded by the proteasome; however, proteasome activity can be inhibited by large protein polymers due to their inability to pass through the narrow proteasome channel; thus, autophagy is stimulated to eliminate those protein complexes [14, 15]. Macroautophagy, microautophagy and chaperone-mediated autophagy (CMA) are the three main forms of autophagy [12]. However, microautophagy is mainly involved in the sequestration of damaged cell organelles through invaginations of the lysosomal membrane, as discussed well in Bao Jin Ku's article [16]. Thus, autophagy lysosome system (ALS) dependent protein degradation is primarily accomplished via macroautophagy and CMA. Proteins were thought to be non-selectively degraded in the lysosome, along with random cytoplasmic components and organelle engulfment, although this notion is now being revisited. Indeed, accumulating evidence has demonstrated that substrate degradation is tightly controlled in the lysosome via autophagy targeting mechanisms that remain far from fully understood in eukaryotic cells.

Autophagy occupies a central position in the maintenance of cellular homeostasis by directing protein degradation, and the process adapts cells to adverse micro-environmental conditions. Accordingly, unraveling more details about autophagic protein degradation pathways may not only improve our understanding of cell metabolism and fate, but may also help us understand a diverse range of human diseases. In this review, we focus on diverse autophagy-mediated protein degradation processes and their regulation.

## THE TARGET PROTEIN FORMS IN SELECTIVE AUTOPHAGY

The ubiquitin proteasome system (UPS) and the autophagy-lysosome system (ALS) are alternative ways to categorize protein degradation based on the functions of the proteins being degraded. It is well known that large protein complexes cannot fit into the narrow proteasomal channels and are thus directed to lysosomal disruption [14, 15].

**Figure 1:**
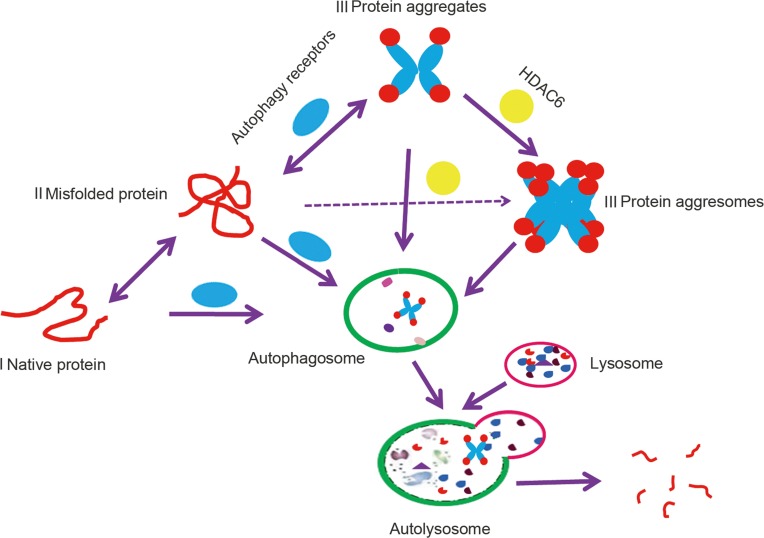
A model of autophagy targeting proteins to degradation in the lysosome Native protein I and soluble misfolded protein II can be degraded by lysosomes or the proteasome; however, when misfolded proteins form insoluble protein aggregates III or aggresomes III, which are organized by HADC6, they are recognized by autophagy receptors and degraded by autophagy.

There are three types of proteins in cells that are degraded by autophagy (Figure 1); the first, cytoplasmic proteins are relatively long-lived and functional [17]. The second, misfolded proteins, are soluble, are monomeric but non-functional and can thus be degraded by the proteasome or lysosome [18]. The last, insoluble misfolded protein complexes include polymers, aggregates and aggresomes [18]. Misfolded proteins can form polymers or aggregates; however, if levels are too excessive to be degraded quickly, histone deacetylase 6 (HDAC6) coupled with dynein motors and microtubules will recruit these misfolded proteins to form an aggresome, or an inclusion body that is localized in the proximity of the microtubule-organizing center (MTOC) [19]. Aggresome formation decreases the toxicity of these proteins and enables cells to focus on essential tasks [19]. Although aggresomes contain different types of misfolded proteins, proteins tagged with the K63 poly-Ub chains were found to be conducive to aggresome formation [20]. Note that ubiquitin as a molecular tag fails to completely determine autophagic protein degradation. Therefore, clearly defining the different types of autophagic protein degradation pathways will improve our understanding of the different mechanisms involved.

## CHAPERONE-MEDIATED AUTOPHAGY (CMA) IN DETERMINING PROTEIN DEGRADATION

**Figure 2:**
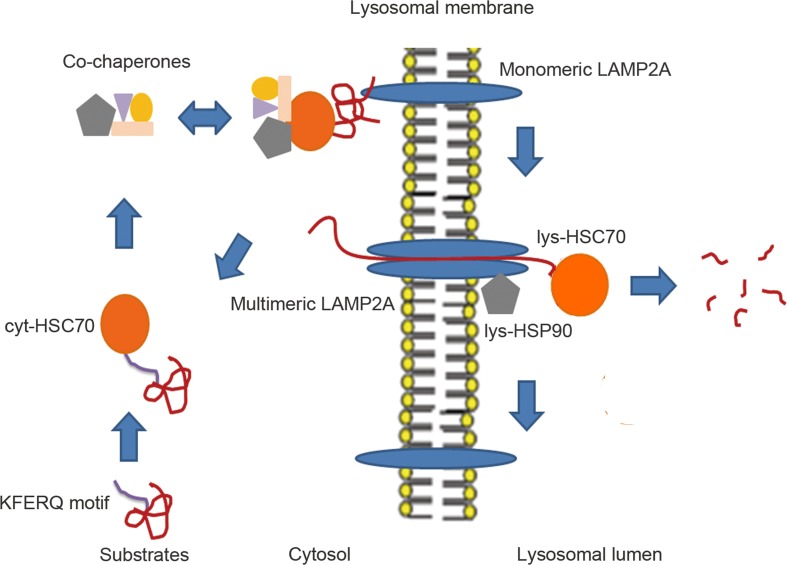
The proposed models of CMA Substrates after partial unfolding or modification via oxidation, ubiquitination and acetylation expose the KFERQ-like motif, which can be recognized by cytosolic Cyt-HSC70. HSC70 co-chaperones are able to form complexes with Cyt-HSC70 and substrates to facilitate unfolding of these substrates and docking onto monomeric LAMP2A, which promotes the multistep organization of LAMP2A into higher-order multimeric complexes. After, Lys-HSC70 can assist the complete unfolded substrates in crossing the LAMP2A complex channel for rapid degradation in the lysosomal lumen. Then, the LAMP2A complex is disassembled into smaller complexes when substrates are no longer present. Lys-HSC90 with LAMP2A at the luminal side of the lysosomal membrane stabilizes this receptor while substrates transit between the multimeric membrane complexes.

Chaperone-mediated autophagy (CMA) is a type of autophagy that degrades soluble or unfoldable proteins in a molecule-by-molecule fashion (Figure 2). In contrast to macroautophagy, CMA can be activated by prolonged starvation to provide amino acids for essential protein synthesis [21]. Heat shock protein 70 (HSC70) and lysosome membrane protein type 2A (LAMP2A) are two key factors that are involved in this process [22].

HSC70 is a member of the heat shock protein 70 family and is located in the cytosol or the lumen of lysosomes [22]. Proteins containing targeting motifs can be recognized by cytosolic HSC70 (Cyt-HSC70) and its co-chaperones such as heat shock protein 40 (HSC40), heat shock protein 90 (HSC90), Bcl-2-associated athanogene1 protein (BAG1) and HSC70-HSP90 organizing protein (HOP), which participate in the unfolding step for the formation of soluble substrate-HSC70 complexes that enable translocation across the lysosomal membrane channels [23]. When substrates are transported to the lysosomal receptor LAMP2A by Cyt-HSC70, lysosomal lumen HSC70 (Lys-HSC70) allows complete passage through the translocation complexes formed by LAMP2A [23]. LAMP2A acts as a receptor for the cytosolic proteins that undergo degradation via CMA and has an N-glycosylated luminal region, a single transmembrane region, and a short cytosolic tail where substrate proteins bind [23]. The monomeric form of LAMP2A preferentially binds to Cyt-HSC70 cargo, but translocation of substrates requires the formation of multimeric LAMP2A complexes, which are stabilized by a form of HSP90 located at the luminal side of the lysosomal membrane [24]. Therefore, LAMP2A serves as a limiting factor in the CMA-lysosome pathway, and unsurprisingly, the exogenous overexpression of LAMP2A has been shown to potentiate the activity of CMA in many validated experiments [25, 26, 27].

### Canonical protein targets of the chaperone-mediated autophagy (CMA) pathway

The criterion for the protein to be a putative CMA substrate is the presence of a peptide sequence that is biochemically related to the KFERQ motif. In brief, the motif is flanked by a glutamine (Q) and contains one acidic residue (D or E), a basic residue (K or R), a hydrophobic residue (F, I, L or V), and a fifth residue that can be basic or hydrophobic but cannot be negatively charged [28]. In particular contexts, glutamine can be replaced by asparagine (N), thereby preserving a similar affinity in the interaction with the chaperone [29]. Based on the presence of this motif, it was speculated that 30% of cytosolic proteins are candidates for CMA [28]; however, the presence of a CMA motif does not guarantee that a protein is strictly degraded by this pathway because post-translational modifications also control this process. Therefore, in the following text, we discuss how oxidative stress and modifications influence protein degradation through the CMA pathway.

### Oxidative stress and post-translational modification-dependent chaperone-mediated autophagy (CMA) pathways

Recent studies have indicated that oxidative stress can activate the CMA pathway and that this potentiation occurs at least partially because oxidized proteins are more easily unfolded to facilitate translocation into the lysosomal lumen. In addition, lysosomes are more active under oxidative stress. For example, the expression of LAMP2A and Lys-HSC70 increased in the lysosomal membrane and lumen during oxidative stress [30]. Moreover, it has been speculated that the oxidation of a histidine may complete a motif that is missing only the negatively charged residue [31]. Therefore, it is rational that 6-AN treatment initiates CMA by providing an oxidative environment within cells [32]. However, a recent study of the CMA degradation of MEF2A provided further insight into the role of oxidative stress in regulation of the CMA pathway. In this study, the degradation of MEF2A via CMA was enhanced under mild oxidative stress (200 μM H2O2); however, the tendency was disrupted under excessive oxidative stress (> 400 μM H2O2), and the opposite results were observed due to obvious lysosomal rupture/permeabilization [33]. However, the alternative possibility that excessive oxidative stress inactivates an existing CMA targeting motif by deamidation and oxidation cannot be ruled out [33]. Above all, oxidative stress can either facilitate or block CMA depending on the extracellular conditions and protein characteristics; therefore, it is plausible that the same oxidative stress may lead to opposite destinations for different proteins.

In addition to oxidative stress and oxidation, other forms of modifications such as phosphorylation, ubiquitination and acetylation can participate in the regulation of CMA. When Phosphoprotein enriched in diabetes (PED) was phosphorylated at Ser104 or Ser116 near the KFERQ-like motifs, its interaction with HSC70 was reduced, thereby precluding its degradation [34]. In contrast to PED phosphorylation, phosphorylated RKIP, ubiquitinated HIF1A and acetylated PKM2 could trigger the degradation of these three proteins by the CMA pathway [35, 36, 37]. The discrepancy may be explained by protein-protein interactions or conformational changes after modification, such that the KFERQ-like motif was either easily masked or exposed to HSC70.

Although the precise mechanism by which the modifications affect the interaction between the protein and HSC70 is not yet clear, many CMA substrates were identified during the last decade and were listed in J. Fred Dice's article [38]. In this review, we summarize the newly demonstrated CMA substrates from recent years and show that the ectopic degradation of these substrates is closely related to many diseases (Table 1). Therefore, further elucidation of the CMA substrates and pathways may have therapeutic potential for treating various diseases.

**Table 1:** Specific proteins known to be degraded by CMA

Protein	Disease	Ref
AF1Q	Acute myeloid leukemia	[27]
Chk1		[39]
EGFR		[40]
GAL3		[41]
HIF1A		[36]
HTT	Huntington Disease	[42]
ITCH		[43]
LRRK2		[44]
MAPT	Alzheimer disease	[45]
MDM2		[46]
MEF2A	Neurodegenerative disorders	[33]
MEF2D	Parkinson disease	[47]
NCOR1	Non-Small cell lung cancer	[48]
p53		[49]
PED	Non-Small cell lung cancer	[34]
PKM2		[37]
PLINs		[50]
PUMA		[51]
RCAN1	Alzheimer disease	[52]
RKIP		[35]
RYR2	Cardiac contractile dysfunction	[53]
SNCA	Parkinson disease	[54]
TARDBP	Neurodegenerative disorders	[55]
UBQLN1		[56]
UCHL1	Parkinson disease	[57]

## DIFFERENT ROLES OF MACROAUTOPHAGY IN SELECTIVE PROTEIN DEGRADATION

Autophagy receptors and the ATG8 family are two primary elements in selective macroautophagic protein degradation. Autophagy receptors bind cargo and the ATG8 family plays a pivotal role in the selective macroautophagy process by promoting the entry of cargo receptors into the autophagy cascade via interaction with the LC3-interacting regions (LIR) of the autophagy receptor [58, 59]. The ATG8 family contains two subfamilies that contain at least seven proteins in humans. The microtubule-associated protein 1 light chain 3 (MAP1LC3 or LC3) group includes MAP1LC3A, MAP1LC3B, and MAP1LC3C, and the γ-aminobutyric acid type A receptor-associated protein (GABARAP) group includes GABARAP, GABARAP-like1 (GABARAPL1), GABARAPL2 and GABARAPL3 [60]. The canonical LIR peptide is the WXXL motif (X stands for any residue), with the tryptophan (W) and leucine (L) residues interacting with two distinct hydrophobic pockets of ATG8 members [61]. Notably, evidence has indicated that the acidic residues such as glutamic acid (E) and aspartic acid (D) that N-terminally precede the LIR motif are indispensable to the LIR-ATG8 interaction, presumably due to its role in potentiating the interaction between the α2 helix of ATG8 and the LIR motif [62]. Therefore, the LIR peptides alone may not guarantee conjugation to the ATG8 members.

**Figure 3:**
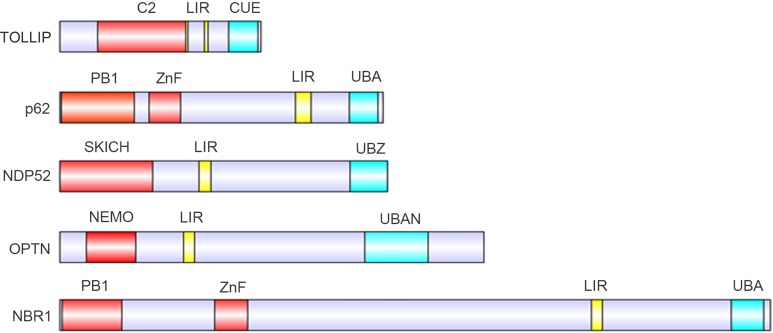
Protein domains of the known autophagy receptors Domain structure of autophagy receptors involved in selective autophagy pathways, containing LC3-interacting motifs (LIR, yellow) and distinct ubiquitin-binding domains (cyan).

To date, several autophagy receptors that mediate protein degradation have been identified, including TOLLIP, SQSTM1/p62, NDP52, OPTN and NBR1. These receptors contain four different ubiquitin-binding domains: the coupling of ubiquitin conjugation to endoplasmic reticulum-associated degradation domain (CUE), the ubiquitin-associated domain (UBA), the ubiquitin-binding zinc finger domain (UBZ) and the ubiquitin binding in ABIN and NEMO domain (UBAN), along with one or two LIR domains (Figure 3) [63, 64].

Selective protein degradation by macroautophagy has gained increasing attention due to increased studies of the roles of SQSTM1 in ubiquitinated protein degradation [65]. Recent experiments have unraveled many details about this process, and it seems that selective protein degradation by macroautophagy is not exclusively ubiquitin-dependent. Proteins can also be degraded by macroautophagy in an ubiquitin-independent manner. In the following sections, we discuss the precise mechanism of ubiquitinated protein degradation and how non-ubiquitinated proteins are involved in selective macroautophagy.

### Ubiquitin-mediated selective macroautophagic protein degradation

**Figure 4:**
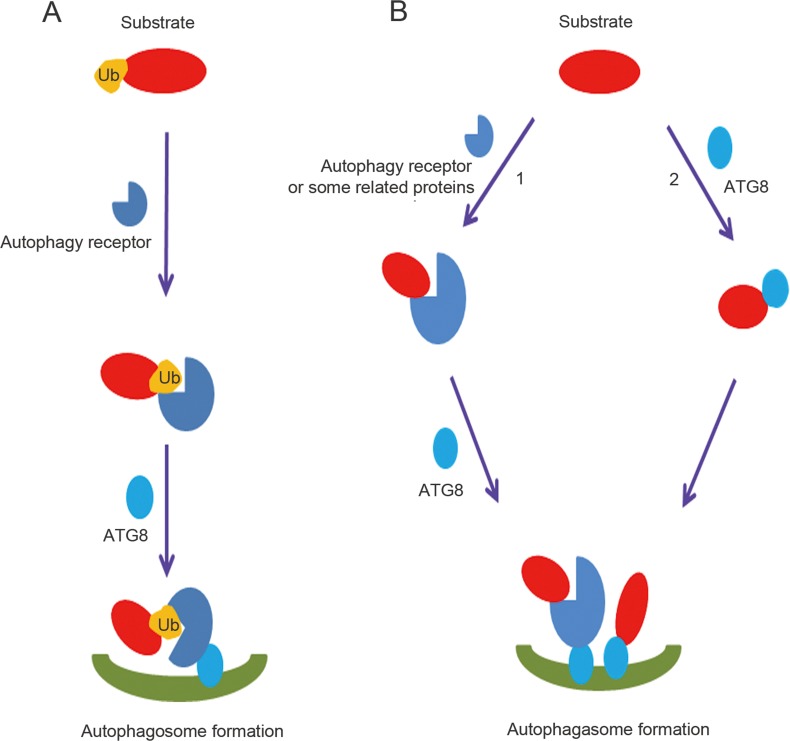
Ubiquitin dependent and independent macroautophagic protein degradation **A.** Overview of ubiquitin-dependent macroautophagic protein degradation. Ubiquitinated proteins are recognized by the ubiquitin-binding domains of autophagy receptors, which then bind ATG8 family members. **B.** Overview of ubiquitin-independent macroautophagic protein degradation. 1. Substrates form complexes with autophagy receptors or related proteins containing a LIR domain independent of ubiquitin and ubiquitin-binding domains, and then are degraded by the lysosome. 2. ATG8 family members can directly recognize LIR domains in proteins and make them substrates for autophagy.

Similar to UPS, there is strong evidence supporting the recognition of ubiquitinated proteins by receptors, which helps to include them in the macroautophagy cascade (Figure 4A) [63]. Proteins can be modified by ubiquitin monomers or ubiquitin chains. Although it was reported that mono-ubiquitination was necessary to degrade active IKK beta by macroautophagy [66], autophagic degradations were most frequently associated with poly-Ub chains [63]. Proteins were able to be tagged by seven types of poly-Ub chains, including K6, K11, K27, K29, K33, K48, and K63 chains. It was reported that K48- and K63-linked proteins were both recognized by p62, NBR1, NDP52 and TOLLIP. However, unlike TOLLIP, the p62, NBR1 and NDP52 proteins prefer binding to K63 chains [63, 64]. Finally, the UBAN domain of OPTN binds specifically to K27 and K48 poly-Ub chain-linked proteins [67].

There are two E1 activating enzymes, 40 E2 conjugating enzymes and more than 500 E3 ligases for ubiquitination in mammals [68]. Distinct substrates can be ubiquitinated by distinct E3 ligases with the assistance of the other two partners, and the E2 conjugating enzymes in concert with E3 ligases determine the type of ubiquitin chain formed on the substrates [69]. Therefore, the E2 enzymes may switch substrates from UPS to ALS due to the low affinity of the autophagy receptor for K48-linked substrates. For example, MDM2 catalyzes the K48-linked ubiquitin chain formation on wild-type p53 in basal conditions and facilitates its degradation by UPS; however, when p53 was mutated, the mutant p53 was prone to aggregate formation [70]. At this time, MDM2 was able to catalyze the formation of its K63 chain with assistance from the E2 enzyme Ubc13, which facilitates recognition of the mutant p53 by SQSTM1, followed by further degradation by ALS [70]. To date, several E3 ligases have been shown to participate in ubiquitin-mediated selective macroautophagy (Table 2), but the specific E2 enzyme and the ubiquitin type that trigger autophagic degradation remain largely unknown. K48, K63 and monoubiquitin chains appear to accelerate the formation of protein inclusion, but only K63-linked proteins are prone to degradation by macroautophagy [20, 79, 80]. However, Esther Wong's study showed that synphilin-1 protein aggregates are susceptible to degradation by basal macroautophagy independent of K63 chain formation [81]; however, when the macroautophagy flux was extremely high due to stress signals such as proteasome inhibition and reactive oxygen species (ROS), macroautophagy would degrade the K63-linked synphilin-1 protein aggresomes derived from the excess aggregates [81]. Therefore, autophagic susceptibility of aggregation-prone proteins may not depend on the nature of the aggregating proteins per se, but on their dynamic properties of the aggregates. Interestingly, NBR1 and SQSTM1 did not interact with these two types of protein complexes. DFCP-1, an effector involved in the nucleation of the autophagosome, was shown to become coated with synphilin-1 aggregates and aggresomes, which may facilitate the inclusion of protein complexes into the ALS [81].

**Table 2:** Specific ubiquitinated proteins known to be degraded by macroautophagy

Protein	Protein types	Autophagy receptor	E3 ligase	Ub chains	Ref
AGO2	Native	NDP52	Unkown	Unkown	[71]
ARTD10	Aggregates	p62	Unkown	Unkown	[72]
DICER1	Native	NDP52	Unkown	Unkown	[71]
Dvl2	Aggregates	p62	VHL	K63	[73]
HIF2A	Aggregates	p62	VHL	Unkown	[74]
HTT	Aggregates	TOLLIP	Rsp5	K48 and K63	[64]
NFKBIA	Aggregates	p62	Unkown	Unkown	[75]
p53	Aggregates	p62	MDM2	K63	[70]
p65	Aggresomes	p62	Unkown	Unkown	[76]
RHOA	Native	p62	Unkown	Unkown	[77]
TFRC	Native	OPTN	Unkown	Unkown	[78]

Moreover, the post-translational modifications of autophagy receptors can influence their interaction with ubiquitinated substrates. The phosphorylation of p62 S403 in its UBA domain by casein kinase 2 (CK2) enhances its binding affinity to ubiquitinated proteins and then promotes the autophagic clearance of the substrate [82]. However, the effect of NBR1 phosphorylation by GSK3 at Thr586 of its LIR domain differed from the above effect of p62 [83]; thus, further investigation should focus on clarifying this discrepancy.

Unlike the ubiquitination in the ALS, deubiquitinating enzymes that negatively regulate autophagic protein degradation in mammals have rarely been reported. To date, only one deubiquitinating enzyme, USP36, has been confirmed. USP36 can inhibit selective macroautophagy by preventing the accumulation of ubiquitinated proteins, but the specific substrates of USP36 (whose targeting to autophagosomes is affected) are not yet known [84]. Therefore, more investigations should be conducted to reveal whether other deubiquitinating enzymes exist that can reverse this type of autophagic protein degradation.

### Ubiquitin-independent selective macroautophagic protein degradation

Substantial evidence has implicated ubiquitin as the culprit responsible for macroautophagic protein degradation. However, recent results argue against the possibility that ubiquitin is the ubiquitous code in selective macroautophagy (Table 3). For example, Yong Tae Kwon's study illustrated that p62 can directly interact with N-terminal arginylation of BiP through its Zinc finger (Znf) domain (residues 122-167) but UBA cannot, indicating that the BiP-p62 interaction is not mediated by ubiquitin [88]. This interaction induces an allosteric conformational change in p62, thereby exposing the PB1 and LIR domains. The PB1 domain promotes self-oligomerization and aggregation of p62; then, BiP, together with other cargoes such as cytosolic misfolded proteins, are engulfed by a lysosome through the p62-LC3 cascade [88]. Therefore, the autophagy receptor could act as an intermediate that directly draws non-ubiquitinated proteins into the macroautophagy flux for degradation through their PB1 or Znf domain. However, some reports have characterized another mechanism by which proteins can directly interact with ATG8 family members through their existing LIR domains. For example, the 502(SHWPLI)507 and 3035(RDWVML)3040 domains in β-catenin and Huntingtin have been confirmed to directly interact with LC3B and GABARAPL1, respectively, to trigger degradation by macroautophagy [89, 92]. The reason that β-catenin and Huntingtin bind different ATG8 family members is that the LIR binding domain in GABARAPL1 is negatively charged; however, the domain in LC3B is positively charged, and GABARAPL1 thus prefers to interact with Huntingtin due to the positively charged residue R3035 adjacent to the RDWVML motif [92]. Similarly, LC3B prefers the interaction with the β-catenin protein partially because of the negatively charged phosphorylated residue S502.

**Table 3:** Specific non-ubiquitinated proteins known to be degraded by macrcoautophagy

Protein	Protein types	Autophagy degradation model	Ref
APP	Native state	APP-AP2-LC3	[85]
AR	Aggregates	AR-p62-LC3B	[86]
BCR-ABL	Native state	BCR-ABL-p62	[87]
BiP	Native state	BiP-p62-LC3B	[88]
CTNNB1	Native state	CTNNB1-LC3B	[89]
FAP1	Native state	FAP1-p62	[90]
Ferritin	Native state	Ferritin - NCOA4- LC3/ GABARAP	[91]
HTT	Native state Aggregates	HTT-GABARAPL1/p62-LC3B HTT-OPTN-LC3	[92,93] [94]
Keap1	Native state	Keap1-p62-LC3	[95]
MAPT	Native state	MAPT-NDP52-LC3	[96]
PrP	Aggregates	PrP-p62-LC3B	[97]
SOD1	Aggregates	SOD1-p62/OPTN-LC3	[94,98]
Src	Native state	Src-c-Cbl-LC3B	[99]
STAT5A	Oligomers	STAT5A-p62-LC3	[100]
TP53INP1	Native state	TP53INP1-LC3/ GABARAP	[101]
VPRBP	Native state	VPRBP-p62-LC3B	[102]

In addition to the direct interaction with an autophagy receptor or ATG8 family member (Figure 4B), proteins such as AP2 and c-Cbl can draw substrates into macroautophagy [85, 99]. The common feature of these two proteins is the lack of an ubiquitin binding domain and the presence of a LIR sequence that can bind to the ATG8 protein members (Figure 4B). For example, c-Cbl was able to interact and target active Src for macroautophagy, independent of its E3 ligase activity [99]. Above all, non-ubiquitinated protein degradation in the lysosome was completed through direct interaction with ATG8, an autophagy receptor, or a protein that functions as an autophagy receptor.

## THE RELATIONSHIP BETWEEN THE DIFFERENT PROTEIN DEGRADATION PATHWAYS

Macroautophagy and CMA are two distinct mechanisms of protein degradation; however, crosstalk between these pathways has been reported. BAG3 coupled with HSC70, which participates in CMA by recognizing the KFERQ motif in substrates, can release HSC70 substrates to the dynein motor complex, thereby mediating aggresome targeting and the macroautophagic degradation of chaperoned substrates [103]. This process recently has been demonstrated and identified as chaperone-assisted selective autophagy (CASA), which requires the involvement of molecular chaperones such as HSC70, HSP22 and the co-chaperones BAG3 and STUB1 [104]. Thus, it is likely that the same substrate may undergo macroautophagy and CMA pathways at the same time; for example, Huntingtin protein has a functional CMA recognition motif KDRVN and a LIR motif WVML, which simultaneously determine its interaction with HSC70 and GABARAPL1 and subsequent degradation via these two methods [42, 92]. Therefore, the actual protein degradation pathway in cells may depend on specific stimuli, cellular context and experimental conditions.

In addition to the crosstalk between different autophagy pathways, autophagy and proteasome pathways have been demonstrated to work complementarily to degrade proteins under some circumstances. For example, when the proteasome system cannot degrade large protein aggregates, HSP90 and Poh1 help the aggregates dissociate from 20S proteasomes while macroautophagy is activated to facilitate clearance of these protein aggregates [105]. Therefore, lysosome and proteasome protein degradation pathways can doubtlessly switch or occur simultaneously in these organelles (Table 4). The target protein conformations or post-translational modifications can determine the specific type of degradation pathway. For example, the E3 ligase Skp2 targets the ubiquitination of the native androgen receptor (AR) and facilitates its proteasomal degradation [120]; however, when AR was mutated via expansion with a polyQ tract and was prone to aggregation, autophagy was initiated to eliminate the mutant AR proteins [86]. The wild-type Tau protein was degraded by the proteasome, but its phosphorylated form was degraded by autophagy, although the mechanisms mediating these processes are unknown [158].

**Table 4:** Specific proteins known to be degraded by lysosome and proteasome

Protein	Degradation type	Ref
ANXA1	CMA and proteasome	[106, 107]
Chk1	CMA and proteasome	[39, 108]
EGFR	CMA and proteasome	[40]
HIF1A	CMA and proteasome	[36, 109]
LRRK2	CMA and proteasome	[44, 110]
MDM2	CMA and proteasome	[46, 111]
MEF2A	CMA and proteasome	[33, 112]
MEF2D	CMA and proteasome	[47, 113]
PED	CMA and proteasome	[34, 114]
PUMA	CMA and proteasome	[51, 115]
RCNA1	CMA and proteasome	[52]
RKIP	CMA and proteasome	[35, 116]
RYR2	CMA and proteasome	[53, 117]
AGO2	Macroautophagy and proteasome	[71, 118]
APP	Macroautophagy and proteasome	[85, 119]
AR	Macroautophagy and proteasome	[86, 120]
ATXN3	Macroautophagy and proteasome	[121, 122]
Bcl-xL	Macroautophagy and proteasome	[123, 124]
BCR-ABL	Macroautophagy and proteasome	[87, 125]
BMAL1	Macroautophagy and proteasome	[126]
Caspase3	Macroautophagy and proteasome	[127, 128]
CDKN1A	Macroautophagy and proteasome	[129, 130]
c-IAP	Macroautophagy and proteasome	[131, 132]
CTNNB1	Macroautophagy and proteasome	[89, 133]
DICER1	Macroautophagy and proteasome	[71, 134]
Dvl2	Macroautophagy and proteasome	[73, 135]
Ferritin	Macroautophagy and proteasome	[91, 136]
HIF2A	Macroautophagy and proteasome	[74]
KRAS	Macroautophagy and proteasome	[137, 138]
LC3A/B	Macroautophagy and proteasome	[139, 140]
NFKBIA	Macroautophagy and proteasome	[75, 141, 142]
p62	Macroautophagy and proteasome	[143]
PARP1	Macroautophagy and proteasome	[144, 145]
PKD2	Macroautophagy and proteasome	[146, 147]
RHOA	Macroautophagy and proteasome	[77, 148]
SIMPLE	Macroautophagy and proteasome	[149]
SNCAIP	Macroautophagy and proteasome	[81, 150]
SOD1	Macroautophagy and proteasome	[94, 98, 151]
Src	Macroautophagy and proteasome	[99, 152]
TFRC	Macroautophagy and proteasome	[78, 153]
TOR1A	Macroautophagy and proteasome	[154]
XIAP	Macroautophagy and proteasome	[155, 156]
HTT	Macroautophagy, CMA and proteasome	[42, 94, 157]
MAPT	Macroautophagy, CMA and proteasome	[45, 158]
p53	Macroautophagy, CMA and proteasome	[49, 70]
SNCA	Macroautophagy, CMA and proteasome	[54, 159]
TARDBP	Macroautophagy, CMA and proteasome	[55]

Moreover, the proteasome and lysosome are believed to degrade misfolded proteins with the help of heat shock proteins, and it was therefore believed that these pathways are both activated to eliminate misfolded proteins that are able to pass through the narrow proteasome channel. Interestingly, the SOD1 G93A mutant was reported to be ubiquitinated by E3 ligase gp78, which targets the protein for proteasome degradation [151]. Haining Zhu and colleagues demonstrated that p62 could also directly bind the SOD1 G93A mutant and mediate its autophagic degradation [98]. This observation highlights that the proteasome and lysosome pathways are biologically relevant and can work together to remove toxic substrates in cells.

## PROTEIN DEGRADATION: THERAPEUTIC OPPORTUNITIES

Prevalent human diseases, including neurodegenerative diseases and carcinomas, have been reported to be attributable to dysfunction of protein degradation; therefore, certain functional elements in ALS or UPS tend to be used as either diagnostic or therapeutic targets in the treatment of these diseases.

A recent finding has indicated that lysosomal proteins LAMP1 and LAMP2 and the autophagy protein LC3B in human cerebrospinal fluid (CSF) may be potential novel biomarkers for Alzheimer disease [160]; however, up to this point, most research has focused on the prognostic or predictive value of autophagy in human cancer. For example, low expression of Beclin1 is predictive of a malignant phenotype and poor prognosis in breast cancer [161], extrahepatic cholangiocarcinoma [162], cervical cancer [163], non-small cell lung cancer [164] and gastric cancer [165]. In contrast, LC3 deficiency is correlated with less aggressive behavior and positive prognostic outcomes in esophageal squamous cell carcinoma [166], oral squamous cell carcinoma [167] and hepatocellular carcinoma [168]. Thus, autophagy-based cancer clinical trials should select autophagy inhibitors or activators that can induce apoptosis or autophagic cell death contingent upon different tumor contexts. It has been acknowledged that autophagy endows established cancer with survival advantages partly via degradation of pro-apoptotic proteins such as caspase3 [127], caspase8 [169] and PUMA [51]. Therefore, chloroquine (CQ) and bafilomycinA1 as lysosomal acidification inhibitors may execute their anti-tumor function by potentiating apoptosis [170, 171]. However, hydroxychloroquine (HCQ) is another CQ analogue that failed in a phase II clinical trial [172], indicating that the use of single-drug therapy may not be sufficient to inhibit autophagy and affect tumor growth. Fortunately, other candidate autophagy inhibitors have surfaced recently, such as the ULK1 kinase inhibitor SBI-0206965 and mTOR activator 3BDO [173, 174], which are probably beneficial for ameliorating autophagy in tumor or cardiovascular diseases. Thus, various autophagy inhibitor combinations or autophagy inhibitors coordinating with other chemicals may provide new therapeutic routes. In addition, in some circumstance, autophagy must be enhanced, particular in neurodegenerative diseases. For example, the inhibition of mTOR by rapamycin removes both soluble and aggregated forms of Aβ, which could improve the cognitive deficits associated with Alzheimer disease [175]. QBP1-HSC70bm peptides, which contain two mutant Huntingtin protein binding domain-polyglutamine binding peptide 1 (QBP1) domains and two HSC70 binding motifs (HSC70bm) reduced mutant Huntingtin protein aggregates and ameliorated the Huntington disease phenotype in a mouse model [176]. However, in some apoptosis-resistant cancers, other non-specific autophagy inducers such as 4-hydroxytamoxifen (OHT) and Bcl-XL-inhibitor Z36 triggered autophagic cell death by K-Ras autophagic degradation and disrupting the interaction between Bcl-XL and Beclin1, therefore playing a significant role in killing tumor cells [137, 177].

Similar to ALS, abnormal UPS, including its proteasomes and proteases, is an indicator and target of numerous human diseases [178]. The anti-tumor and anti-inflammatory properties of Bortezomib, the representative of proteasome inhibitors, have been widely approved [179]. However, drug resistance caused by proteasome inhibitors, particularly in solid tumors, has also led to an interest in identifying alternative targets that function upstream of the proteasome degradation machinery. Based on their diversity, E3 ligases and deubiquitinases (DUBs) represent a potential wealth of incompletely tapped targets for drug development. For instance, there are at least five inhibitors of HDM2 (the E3 ligase of p53) that are in phase I clinical trials, which leads to cell cycle arrest and apoptosis in cancer cells with wild-type p53 [180]. USP7 stabilizes the E3 ligase MDM2 due to its deubiquitinating activity, and hence indirectly leads to enhanced p53 proteasomal degradation. Consequently, a selective inhibitor of USP7, P5091, recently demonstrated anti-tumor activity in *in vitro* and *in vivo* models of myeloma [181].

Proteasome inhibitors such as Bortezomib and DUB inhibitor PR-619 can trigger autophagy, although the mechanisms of these activities are not yet fully understood [182, 183]. In contrast, UPS can also be induced via the inhibition of autophagy, which has been proved by increased proteasomal activities and the upregulation of proteasomal subunits [184]. Therefore, the combined use of autophagic and UPS inhibitors which fully block protein degradation avenues may suppress tumor growth more significantly than either agent in isolation [185]. Excitingly, a phase I trial combining bortezomib and HCQ demonstrated the feasibility of this approach in treating multiple myeloma [186].

## CONCLUDING REMARKS AND FUTURE PERSPECTIVES

Autophagic degradation has been demonstrated to be selectively and precisely regulated. However, details regarding this type of degradation pathway remain largely unknown. Ubiquitination has been shown to accelerate autophagic protein degradation by interacting with the ubiquitin-binding domains in autophagy receptors. Interestingly, recent studies showed that, compared with non-arginylated BiP and non-acetylated Huntingtin proteins, arginylated and acetylated proteins strongly bind p62 to undergo degradation by autophagy, though the precise mechanisms remain unknown [88, 93]. Thus, post-translational modifications other than ubiquitination that regulate autophagic protein degradation should be further elucidated.

Long noncoding RNAs (lncRNAs), defined as transcripts longer than 200 nucleotides, are a currently popular study topic in research due to their far-ranging functions in regulating protein-protein and protein-RNA interactions [187, 188]. Therefore, it is no surprise that lncRNA can influence proteasomal protein degradation by disrupting the interaction between substrates and their E3 ligases [189]. Due to the similarity between UPS and ALS, it is possible that some lncRNAs also participate in the regulation of autophagic protein degradation by interfering with the interaction between substrates and autophagy receptors; however, there is no experimental evidence to support this theory to date.

Ectopic accumulation and distribution of functional proteins are adverse to human health. Although autophagic removal of insoluble toxic proteins or oncogenic proteins could relieve neurological or tumorous symptoms respectively, specific and effective targeting by autophagy drugs is insufficient compared with numerous UPS intervening agents. Therefore, the questions of whether any drugs that can selectively modulate protein and autophagy receptor interactions or whether E3 ligase inhibitors or DUB inhibitors can control the autophagic degradation of certain protein targets remain to be further determined. Further investigation of autophagic protein degradation mechanisms may help elucidate these questions and provide more unique therapeutic strategies to treat human diseases.
